# Randomized Trial of Safety and Effectiveness of Chlorproguanil-Dapsone and Lumefantrine-Artemether for Uncomplicated Malaria in Children in The Gambia

**DOI:** 10.1371/journal.pone.0017371

**Published:** 2011-06-07

**Authors:** Samuel Dunyo, Giorgio Sirugo, Sanie Sesay, Cyrille Bisseye, Fanta Njie, Majidah Adiamoh, Davis Nwakanma, Mathurin Diatta, Ramatoulie Janha, Fatou Sisay Joof, Beth Temple, Paul Snell, David Conway, Robert Walton, Yin Bun Cheung, Paul Milligan

**Affiliations:** 1 Medical Research Council Laboratories, Banjul, The Gambia; 2 Faculty of Epidemiology and Population Health, London School of Hygiene and Tropical Medicine, London, United Kingdom; New York University School of Medicine, United States of America

## Abstract

**Background:**

Chlorproguanil-dapsone (Lapdap), developed as a low-cost antimalarial, was withdrawn in 2008 after concerns about safety in G6PD deficient patients. This trial was conducted in 2004 to evaluate the safety and effectiveness of CD and comparison with artemether-lumefantrine (AL) under conditions of routine use in G6PD normal and G6PD deficient patients with uncomplicated malaria in The Gambia. We also examined the effects of a common genetic variant that affects chlorproguanil metabolism on risk of treatment failure.

**Methods:**

1238 children aged 6 months to 10 years with uncomplicated malaria were randomized to receive CD or artemether-lumefantrine (AL) and followed for 28 days. The first dose was supervised, subsequent doses given unsupervised at home. G6PD genotype was determined to assess the interaction between treatment and G6PD status in their effects on anaemia. The main endpoints were clinical treatment failure by day 28, incidence of severe anaemia (Hb<5 g/dL), and haemoglobin concentration on day 3.

**Findings:**

One third of patients treated with AL, and 6% of patients treated with CD, did not complete their course of medication. 18% (109/595) of children treated with CD and 6.1% (36/587) with AL required rescue medication within 4 weeks, risk difference 12% (95%CI 8.9%–16%). 23 children developed severe anaemia (17 (2.9%) treated with CD and 6 (1.0%) with AL, risk difference 1.8%, 95%CI 0.3%–3.4%, P = 0.02). Haemoglobin concentration on day 3 was lower among children treated with CD than AL (difference 0.43 g/dL, 95% CI 0.24 to 0.62), and within the CD group was lower among those children who had higher parasite density at enrolment. Only 17 out of 1069 children who were typed were G6PD A- deficient, of these 2/9 treated with CD and 1/8 treated with AL developed severe anaemia. 5/9 treated with CD had a fall of 2 g/dL or more in haemoglobin concentration by day 3.

**Interpretation:**

AL was well tolerated and highly effective and when given under operational conditions despite poor adherence to the six-dose regimen. There were more cases of severe malaria and anaemia after CD treatment although G6PD deficiency was uncommon.

**Trial Registration:**

Clinicaltrials.gov NCT00118794

## Introduction

Chlorproguanil-dapsone (CD) was developed as a low-cost treatment for uncomplicated malaria [1]. CD was withdrawn in 2008 due to concerns about safety in G6PD-deficient patients [2]. Initial trials showed that CD was well tolerated and effective [3]–[5]. In a phase 3 trial in children, 96% showed clinical and parasitological cure by day 14 after the start of treatment, but children with G6PD A- deficiency were more likely to have a fall of haemoglobin of 2 g/dL or more by day 3 if they had been treated with CD, and this association was more marked if baseline temperature was high [6]. Following this trial, a series of studies was planned to evaluate the safety and effectiveness of CD in operational settings in order to evaluate the extent to which the risks associated with the use of the drug in settings without G6PD screening might outweigh the benefits to malaria treatment [7] of which the present study conducted in 2004 was the only one to be completed. More recently, two trials of CD combined with artesunate (CDA) [8.9], showed clearly that malaria patients with G6PD A- deficiency who were treated with CDA were more likely to develop severe anaemia following treatment than G6PD deficient patients treated with SP+AQ [8] or AL [9] and in light of these findings further development of CDA (Dacart) was stopped and CD (Lapdap) was withdrawn by GSK [2].

In The Gambia the first line treatment for uncomplicated malaria was changed from chloroquine (CQ) to sulfadoxine-pyrimethamine plus CQ in 2004 following the finding of 28% clinical failure rate in children treated with CQ in 2003 (M Jawla, unpublished data), and artemisinin combination therapy with artemether-lumefantrine (AL) was adopted in 2007. The present trial was conducted in 2004 to evaluate the safety of CD and its effectiveness in operational settings in comparison with AL, and to compare effects of these drugs on anaemia in G6PD normal and G6PD deficient patients with uncomplicated malaria. We also examined the effects of a common genetic variant that affects chlorproguanil metabolism on risk of treatment failure. We aimed to evalue the alternative regimens under conditions as similar as possible to those of routine use, when doses given at home are not supervised by trial staff, since adherence may affect outcome [10] and may vary with drug acceptability and regimen, and absorption of lumefantrine is affected by diet which is often closely monitored in trials.

## Methods

### Study population

Children aged 6months to 10 years presenting with fever or recent history of fever or other symptoms suggestive of malaria were screened at three health centres in The Gambia from October to December 2004 (Farafenni Maternal and Child Health Clinic, which serves the rural town of Farafenni; Njaba Kunda, a mission clinic in a rural village; and Brikama Health Centre, a Government health centre serving a large urban population). Axillary temperature was measured using a digital thermometer, the patient was weighed, and a finger prick blood sample was collected for measurement of packed cell volume, haemoglobin concentration and malaria microscopy. The study was explained by a field assistant who sought written consent. Patients were then assessed by a nurse or clinician (a nurse in 2 sites, a clinician in one site), and were invited to enrol into the study if they had monoinfection with *P. falciparum* with density between 500–200,000 parasites/µL, PCV ≥20%, no serious concomitant illness, and a parent or guardian had given written consent. Patients were excluded if there were signs or symptoms of severe malaria (unable to drink or feed, repeated vomiting, convulsions with the present illness, lethargic or unconscious state, unable to sit or stand, or parasite density ≥200,000 asexual parasites/µL of blood; if they had severe malnutrition, defined as 3SD below weight for height, clinically evident concomitant disease, or history of allergy to the study medications, and if travel outside the study area was planned before the end of the follow-up period. Patients who did not satisfy the inclusion criteria were treated according to national guidelines.

### Randomization and treatment allocation

The randomization list was produced with Stata version 8 (Statacorp Texas) using permuted blocks of size 10. Eligible children were allocated to receive three daily doses of CD (chlorproguanil 2.0 mg/kg and dapsone 2.5 mg/kg body-weight (manufactured by Wülfing Pharma GmbH, D-31028 Gronau, Germany and supplied by GlaxoSmithKline, UK), or a six-dose course of AL (20 mg artemether and 120 mg lumefantrine; manufactured by Beijing Novartis Pharma Ltd, Beijing, China for Novartis Pharma AG, Basle, Switzerland and procured through the World Health Organization (WHO, Geneva). Patients who were enrolled were issued with an opaque envelope, labelled with the next sequential study number, containing the treatment allocation and an appointment card for follow-up visits pre-printed with the same study number. The children were then referred to a drug dispenser, a nurse who took no part in patient assessment, who opened the randomization envelope and supervised administration of the first treatment dose. Dosages of both medications were weight-determined according to manufacturers' weight cut-off instructions and were dispensed in their original blister packs. For AL, patients received the World Health Organization (WHO) weight-specific blister packs as follows: 10.−14.9 kg: 1 tablet per dose;15.0−24.9 kg: 2 tablets; 25.0−34.9 kg: 3 tablets, and ≥ 35 kg: 4 tablets. The packs contained pictorial instructions on the regimen schedule. CD was supplied in two formulations: Lapdap 15/18.75, tablets containing 15 mg chlorproguanil hydrochloride and 18.75 mg dapsone are for use in infants and young children, white capsule-shaped tablets with a break line on one side for easy dispensing as half tablet dose and marked CD15 on the other side, and Lapdap 80/100 tablets containing 80 mg chlorproguanil hydrochloride and 100 mg dapsone, pink capsule-shaped tablets indicated for the treatment of older children and adults. Both tablet formulations have a break line on one side and are marked with the strength (CD15 or CD80) on the other side. CD was given according to weight. Children under 16 kg received Lapdap 15 tablets (<8 kg, one tablet; 8–11.9 kg, 1.5 tablets; 12–15.9, 2 tablets). Children 16 kg and over received Lapdap 80 tablets (16–20.9 kg, 0.5 tablet; 21–39.9 kg, 1 tablet).

The drug dispenser explained the number and timing of treatment doses to be given, and the first dose of medication was given by the parent/carer at the health centre under direct observation. Patients were observed for 30 minutes, and children who vomited during the observation period had their doses repeated. If the child vomited both the first and replacement medication then a rescue medication was given (parenteral chloroquine plus SP to be taken later). In addition to the study drugs, oral paracetamol (10 mg/kg) was given in the clinic and additional doses were given to parents for administration to the children three times a day at home. Iron tablets (0.5 tablet per day for children under 5 and 1 tablet per day for older children) to be taken daily for 2 weeks, were given to children whose PCV at enrolment was less than 30%.

### Follow-up

The parent or carer was encouraged to bring the child back to clinic if the condition did not improve or if they had any concerns about the child's health. Subsequent antimalarial treatment doses were to be given at home unsupervised. The parent/carer was requested to return the patient to the clinic on days 14 and 28 after treatment for clinical evaluation and blood sampling for measurement of haemoglobin concentration, PCV, malaria microscopy and parasite genotyping. Children were visited at home on the day after the regimen should have been completed (day 3) to ask about compliance with the treatment regimen and adverse events, and to check for signs of anaemia and haemolysis (pallor and jaundice). Blister packs were inspected to count left over medication, and a finger prick blood sample was collected from the child to measure haemoglobin concentration. Patients with severe anaemia (defined as Hb ≤5 g/dl) or Hb that had fallen by ≥2 g/dl from Day 0 measurement were promptly referred to the clinic for clinical assessment and treatment (transfusion for severely anaemic children, and iron supplementation for those with moderate anaemia). Nurses and drivers were placed on 24-hour emergency duty at the health centres/hospital where the study was sited to handle emergencies.

### Nested case control study

A nested case control study was used to determine whether CD is less effective in children who when screened carried parasites with the triple dihydrofolate reductase (DHFR) mutations encoding isoleucine at position 51 (51I), argentine at position 59 (59R), and asparagines at position 108 (108N). Cases were children treated with CD who had detectable parasitaemia by day 28, and controls were children from the same treatment group who remained *P. falciparum* negative up to day 28, frequency matched by centre. In a secondary analysis, a subset of these cases, defined as children with parasitological treatment failure by day 14, was used. In addition, for the CD treatment failures, parasites were typed from samples collected on the day of failure, to detect any increase in the proportion of children carrying parasites with the resistant mutations. The same cases and controls were used to investigate the possible association between CYP2C19 genotype and CD treatment failure. This cytochrome P450 enzyme is responsible for activating chlorproguanil. The *2 allele is a frequently-occurring functional CYP2C19 variant characterised by a G/A polymorphism in exon 5 which creates an aberrant splice site. The resulting change in reading frame of the mRNA introduces a premature stop codon which results in a truncated and non functional protein [11], [12].

### Laboratory methods

At screening and at follow-up visits, two thick blood films were prepared, one was stained with Field Stain and read immediately for malaria diagnosis for enrolment, the second was dried overnight and stained with Giemsa for definitive microscopy. One hundred high power field fields (HPFs) were read before a slide was declared negative. Parasite density was determined assuming one parasite per HPF equivalent to 500 parasites/µL blood [13]. Haemoglobin concentration was measured from finger prick blood samples using a portable haemoglobin machine (HemoCue AB, Ängelholm, Sweden); samples of blood were collected into heparinised capillary tubes and spun using a micro-haematocrit centrifuge (Hawksley, England) to measure PCV.

Using DNA extracted from blood spots, the DHFR codon 51, 59 and 108 alleles were genotyped by polymerase chain reaction (PCR) amplification followed by analysis restriction fragment length polymorphism (PCR-RFLP) [14].

G6PD genotype was estimated using an ARMS PCR technique to detect the two A-associated mutations in G6PD enzyme, an A to G transition at position 376, encoding the B (normal) to A change and a G to A transition at position 202, encoding the specific A- change [15].

CYP2C19 typing was performed using the Taqman real time PCR technique to detect the *2 allele by the presence or absence of the A/G polymorphism [16]. We also typed 100 healthy children for the *3 allele which also results in a non functioning allele and did not find it in our population.

### Endpoints, sample size, and statistical analysis

The primary endpoint was clinical failure by day 28 and change in haemoglobin between day 0 and day 3. To demonstrate that the treatments were at least as good as each other assuming a non-inferiority limit of 3% with at least 80% power at 0.025 significance level, 1180 patients were required to be randomized, allowing for 10% loss to follow up. Since patients with glucose-6-phosphate dehydrogenase (G6PD) A- deficiency are more susceptible to the haemolytic effects of the dapsone component of CD we were interested in the interaction between G6PD status and treatment drug in their effects on haemoglobin concentration. We estimated that a trial of this size would have 80% power (with a 5% significance level) to detect an interaction between treatment drug and G6PD status if the average fall in Hb by day 3 was 2 g/dl in normal patients in both drug groups, and if the fall is 3.1 g/dl or more in G6PD patients who took CD. Other secondary endpoints were the overall clinical and parasitological failure rate (including detection of parasitaemia after day 3), severe anaemia (Hb≤5 g/dL) at any time during the 28-day follow-up, and a drop to 2 g/dL or more below the screening value at any time during the 28 days. An analysis plan was written before the treatment code was linked to the study database.

95% CI for risk differences were computed using a generalised linear model with an identity link to adjust for centre, in Stata 8.2 (StataCorp, College Station, Texas, USA). Primary analysis was according to protocol (excluding individuals with no follow-up data, and according to the treatment drug actually received, but not excluding children who did not complete the regimen). Changes in Hb concentration from baseline were compared between groups by using analysis of covariance, adjusting for the baseline value as a covariate.

The study protocol was written following the principles of the Declaration of Helsinki and was reviewed by the MRC Scientific Coordinating Committee (SCC) and approved by the joint Gambian Government/MRC Ethics Committee. Meetings were held in study villages to explain the aims of the study. At the clinics, the aims and procedures and potential risks were explained to the mother or carer of children who had screened positive for malaria, in their usual language, and their signed consent was sought before the child was enrolled. A Data Safety Monitoring Board was set up for the trial and an experienced physician, who was not part of the trial team, was appointed as local safety monitor to advise on adverse events. The protocol for this trial and supporting CONSORT checklist are available as supporting information; see Checklist S1 and Protocol S1.

## Results

### Participant flow

**Figure 1:**
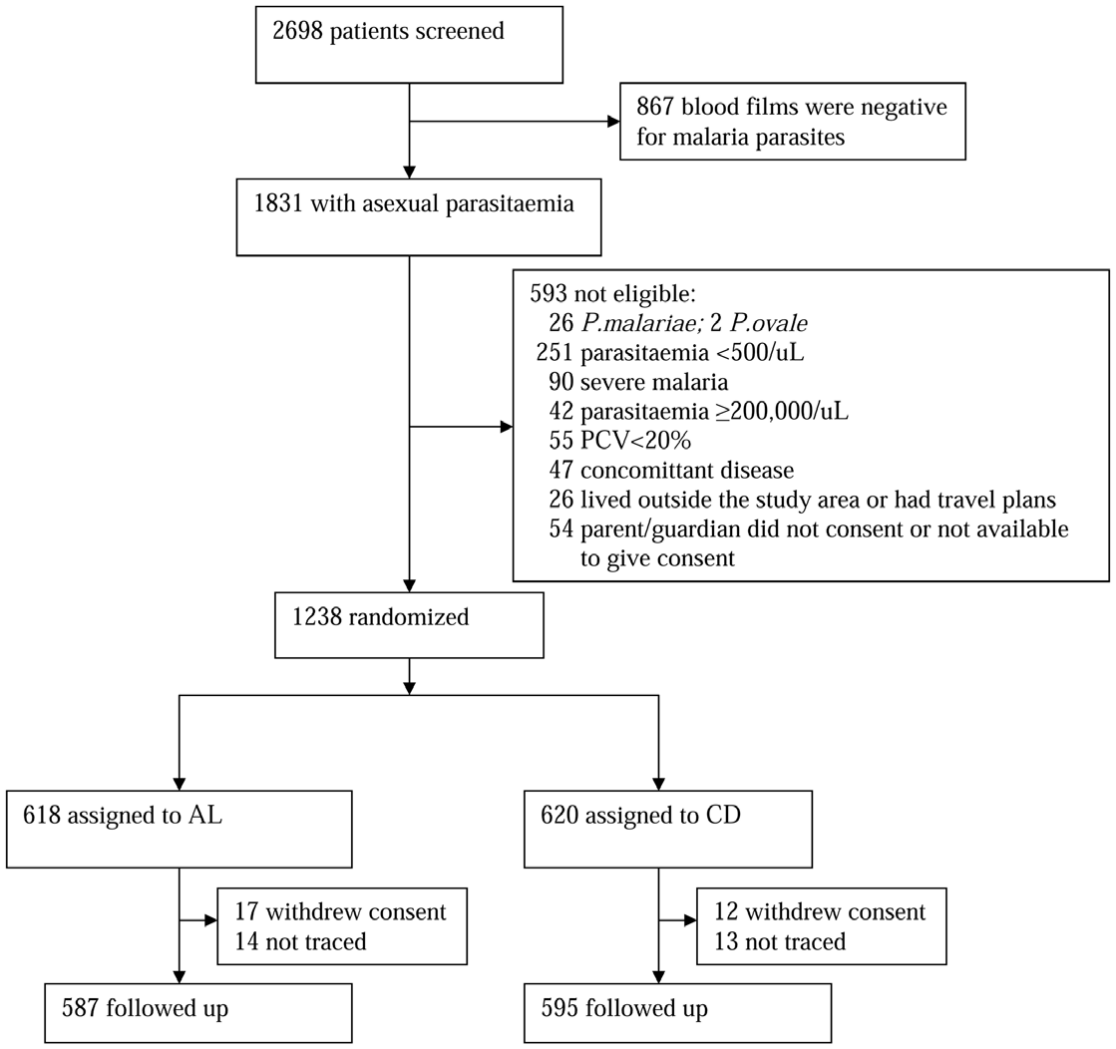
Trial profile.

2698 children presenting with fever, history of fever or other symptoms suggestive of malaria were screened, 1831 (68%) were positive for *P.falciparum*, 26 for *P.malariae* and 2 with *P.ovale*. Of those with *P falciparum* monoinfection, 1238 were enrolled and randomized to receive AL or CD. Of the remaining 593 who did not satisfy the inclusion criteria, 251 (42%) had low density asexual parasitaemia (<500 parasites/µL), 90 (15%) had signs and symptoms of severe malaria, 55 (9.3%) were severely anaemic (PCV <20%). 54 (9.1%) patients were not enrolled because parents/guardians did not consent to their participation in the study, 47 had concomitant disease, 42 had very high density asexual parasitaemia (>200,000 parasites/µL). A total of 1122 (90.6%) patients (560 AL, 562 CD) were seen on day 28. The trial profile is shown in Figure 1.

4 children randomised to AL who received CD in error and 1 child randomised to CD who received AL, were analysed according to the treatment received. 56 patients with no outcome data: 29 withdrew consent to be followed up (17 Co-artem, 12 CD) and 27 were not contactable due to wrong address or residence across the Gambian border (14 AL, 13 CD) were excluded from the analysis. Table 1 shows baseline characteristics of both groups. 234 mainly younger patients were enrolled from Brikama, the costal site (mean age (standard deviation, SD) 3.9 years, (2.3)) compared with 466 in Farafenni (5.2 years (2.7) and 482 in Njaba Kunda (5.2 years (2.5), the upcountry sites. Farafenni centre had a greater proportion of Wolof ethnic patients (22.3%) compared with Brikama (3.4%) or Njaba Kunda (2.3%). Geometric mean density of parasitaemia was higher in Njaba Kunda (15 359 parasites/µL of blood) compared with Brikama (24 299/µL) or Farafenni (30 313/µL.). The prevalence of the G6PD A- genotype in the patients was 5.2% in Farafenni compared with 3.7% in Brikama and 3.1% in Njaba Kunda. Patients spent on average 4 days at home before reporting to the health centre in Brikama compared with 3 days at Farafenni or Njaba Kunda. The CYP2C19 genotype groups were in Hardy-Weinberg equilibrium in the study population (X^2^[1 df] = 0.148, P = 0.7008) with an allele frequency of 0.168.

**Table 1:** Characteristics of patients at enrolment.

Variable	AL (n = 618)	CD (n = 620)
Gender Male:Female	281 (45%):337 (55%)	329 (53%):291 (47%)
Age (in months) mean (range)	60 (6–132)	58 (6–120)
Ethnic group: Mandinka	409 (66%)	404 (65%)
Fula	80 (13%)	73 (12%)
Wollof	79 (13%)	66 (11%)
Others	50 (8.1%)	77 (12%)
Temperature (°C) mean (range)	37.9 (35.1–40.6)	37.9 (35.0–40.7)
Geometric mean parasite density per µL (interquartile range)	21670 (9000–65000)	23007 (9000–71500)
Gametocyte prevalence	20 (3.2%)	35 (5.7%)
Days since onset of symptoms mean (interquartile range)	2.7 (2–3)	2.9 (2–3)
Haemoglobin g/dL mean (range)	10.1 (4.8–15.6)	10.1 (5.3–14.5)
Packed cell volume % mean (range)	31.5 (20–84)	31.3 (15–42)
G6PD A- (hemizygous boys and heterozygous and homozygous girls)*	25/532 (4.7%)	24/537 (4.5%)
Hemizygous males	7	9
Heterozygous Females	17	15
Homozygous females	1	0
Centre: Farafenni	252 (41%)	252 (41%)
Centre: Njaba Kunda	242 (40%)	243 (40%)
Centre: Brikama	124 (20%)	125 (20%)

*G6PD genotype was available for 1069 individuals.

### Adverse events

There were 44 serious adverse events. 13 children were admitted with severe malaria (11 in the CD group, and 2 in the AL group); 23 children had severe anaemia (Hb<5), 17 (2.9%) in the CD group and 6 (1.0%) in the AL group (risk difference 1.8%, 95%CI 0.3% to 3.4%) and 8 children were hospitalized with other conditions, not thought to be related to treatment (5 in the CD group and 3 in the AL group). There were no deaths.

Of the 23 severe anaemia cases, 19 occurred on or before day 3 (15 in the CD group and 4 in the AL group). Children were given iron and folate to be taken at home. Although transfusion was recommended for 10/23 children, only 2 of these were transfused, parents of the other 8 refused. All 23 severe anaemia cases were seen again on day 14 or 28 and their Hb found to have improved (range 7.6 g/dL–11.7 g/dL). One child who had Hb of 5.3 g/dL, and therefore not severely anaemic according to our definition, was brought back to the clinic on day 2 was transfused. G6PD genotype was determined for 1069/1238 children. The frequency of the A- allele was 3.1% (95%CI 2.3% to 4.1%), the genotypes of boys and girls were in Hardy-Weinberg equilibrium (X^2^ [2] = 0.45, P = 0.797). Among G6PD normal children, severe anaemia was detected in 2/507 treated with AL and 12/513 treated with CD. Among the 49 G6PD A- individuals, 1/24 treated with AL and 3/24 treated with CD developed severe anaemia; among hemizygous boys, 2/9 in CD group and 1/7 in the AL group developed severe anaemia; 5/9 in the CD group had a fall in haemoglobin of 2 g/dL or more by day 3, compared to 1/6 in the AL group (Table 2).

**Table 2:** Incidence of severe anaemia after treatment according to G6PD genotype.

G6PD genotype	Incidence of severe anaemia		% with drop in Hb by day 3 of 2 g/dL or more from baseline	
	AL	CD	AL	CD
Normal	2/507 (0.39%)	12/513 (2.3%)	152/491 (31%)	202/495 (41%)
Heterozygous girls	0/17 (0%)	1/15 (6.7%)	6/17 (35%)	7/15 (47%)
Homozygous girls	0/1	-	1/1	-
Hemizygous boys	1/7 (14%)	2/9 (22%)	1/6 (17%)	5/9 (56%)
Not typed	3/82 (3.7%)	2/83 (2.4%)	17/79 (22%)	27/74 (36%)

Of the 13 severe malaria cases, 7 occurred in the first 3 days, all in the CD group. G6PD genotype was obtained for 12 of these cases, all were normal.

4 children who received AL and 6 who received CD vomitted the first and repeat dose of study medication on day 1 and were given rescue treatment.

On day 3, 93 of 601 (15.5%) parents/carers of patients in the CD group and 25 of 606 (4.1%) in the AL group said their children were still unwell, the most commonly reported symptoms were fever, headache, gastrointestinal complains (abdominal pain, anorexia, and diarrhoea), and cough.

### Treatment outcome

109/595 children (18%) in the CD group and 36/587 (6.1%) in the AL group required rescue treatment within 4 weeks, risk difference adjusted for centre 13% (95%CI 8.9% to 16%). By day 14, the corresponding rates were 5.8% and 0.9% (Table 3).

**Table 3:** Treatment failure and anaemia outcomes by treatment group: percentage for failures and anaemia; mean (g/dL) for haemoglobin.

Variable	AL (n = 587)	CD (n = 595)	Difference (95% CI)*	P-value*
Clinical failure by day 14	0.9%	5.8%	5.1% (3.1,7.2%)	<0.001
Clinical failure by day 28	6.1%	18.3%	12.6% (8.9,16.2%)	<0.001
Parasitological failure by day 14	2.2%	8.5%	6.5% (4.0,9.1%)	<0.001
Parasitological failure by day 28	12.9%	38.4%	25.6% (20.9,30.4%)	<0.001
Anaemia drop of 2 g/dL or more from baseline	8.5%	11.8%	1.7% (−0.2,3.6%)	0.072
Severe anaemia (Hb<5 g/dL) by day 28	6 (1.0%)	17 (2.9%)	1.8% (0.3%,3.4%)	0.022
Severe malaria by day 28	2 (0.3%)	11 (1.8%)	1.5% (0.3%,2.7%)	0.0129
Mean Hb g/dL day 3#	8.8	8.4	−0.35 (−0.50,−0.20)	<0.001
Mean Hb g/dL day 14#	10.3	10.1	−0.19 (−0.37,−0.01)	0.039
Mean Hb g/dL day 28#	10.9	10.6	−0.27 (−0.45,−0.10)	0.002
Serious adverse events	11 (1.9%)	33 (5.6%)	3.7% (1.5%−5.8%)	0.0009

*Adjusted for centre.

# differences adjusted for Hb on day 0.

Clinical and parasitological failures at days 14 and 28 were significantly higher in the CD group compared with AL treatment (p<0.001). There was an increase in mean haemoglobin from baseline by day 28, with values significantly higher in the AL group compared with CD treatment. The incidence of anaemia by day 28, defined as haemoglobin <5 g/dl, was higher in the CD group.

### Interaction between treatment group and G6PD status

Mean haemoglobin concentration fell after treatment in both groups, with values in the CD group lower on day 3 than those in the AL group (a difference, after adjustment for baseline, of 0.34 g/dL, 95% CI 0.18 to 0.49, p<0.001). To determine whether CD treatment increased the risk of anaemia among G6PD deficient patients, the interaction between treatment group and G6PD status in their effects on Hb concentration on day 3 needs to be examined. G6PD genotype was determined for 1069 of 1238 (86%) children. Boys carrying the A- allele, and girls homozygous or heterozygous for the A- allele, were identified and classified by DNA genotyping. Hb concentration on day 3 was lower in G6PD A- than in G6PD wild type children, and was lower in children treated with CD than those who received AL, but the number of A- children was too small to evaluate the strength of interaction. When the analysis was limited to G6PD A- deficient hemizygous boys and to the one homozygous girl identified, (thus excluding heterozygous girls who may not be deficient) similar results were obtained; in this subgroup, Hb was slightly lower in the CD group at baseline (9.3 g/dL on day 0 compared to 10.4 g/dL in the AL group); on day 3 the corresponding mean values were 7.2 g/dL and 8.6 g/dL.

### Effect of baseline parasite density

Children with higher parasite densities on day 0 were at increased risk of anaemia after treatment. This effect was evident in both drug groups at day 3 and day 14, and on day 3 appeared to be more marked in the CD group (for anaemia on day 3, the interaction between treatment group and parasite density group (above or below the median value 28000 parasites/µL) is 0.41/dL, 95%CI 0.10 to 0.71, P = 0.009), Table 4. Higher parasite density was associated with higher temperature at screening, but screening temperature was not independently associated with anaemia.

**Table 4:** Haemoglobin concentration (g/dL) on day 3 after treatment in relation to G6PD status and parasite density at enrolment.

	Mean Hb on day 0 g/dL (no. of children)		Mean Hb on day 3 g/dL		Difference in day 3 Hb between groups (AL-CD), (95%CI), adjusted for baseline*
*G6PD genotype:*	AL	CD	AL	CD	
G6PD normal	10.2 (505)	10.1 (509)	8.9	8.5	0.34^a^ (0.18,0.50)
A- hemizygous boys and heterozygous and homozygous girls	9.8 (24)	9.7 (24)	8.3	7.8	0.32^b^ (−0.40,1.0)
A- hemizygous boys and homozygous girls	10.4 (7)	9.3 (9)	8.6	7.2	0.55^c^ (−0.69,1.8)
*Parasite density at enrolment:*					
Low: <median	10.0 (259)	10.1 (257)	8.9	8.8	0.12^d^ (−0.08,0.33)
High: >median	10.3 (255)	10.2 (260)	8.8	8.2	0.56^e^ (0.36,0.76)

*Estimate of b-a, representing the interaction between G6PD status and treatment, is −0.04 g/dL, 95%CI −0.78 to +0.69, P = 0.907. If the heterozygous girls are excluded, (comparison c-a) the estimate is 0.21 g/dL (−1.1 to 1.5) P = 0.755. Estimate of e-d representing the interaction between treatment group and parasite density group is 0.41 g/dL (0.10 to 0.71) P = 0.009.

### Adherence to treatment regimen

Of children in the AL group whose mothers were interviewed about compliance, 402/600 (67%) took all 5 doses at home, 190 (32%) took some but not all the recommended doses at home, 7 (1.2%) took no medication at home. There was no association of compliance with outcome (data not shown). When blister packs were inspected 186/591 (31%) of those whose packs were seen had left-over medication. Of 599 children in the CD group with compliance information, 562 (94%) took all 3 doses, 12 (2%) took no doses at home, 25 (4%) took only 1 dose at home. The poorer compliance with the AL regimen primarily reflects the larger number of doses. Again no association of compliance with outcome was evident. 51/568 (9%) whose blister packets were inspected had left-over medication. The most common reason given for not giving treatments at home was that the mother forgot or did not understand the regimen.

### Nested case control study

Of 229 cases, 8 individuals were excluded because they did not receive CD: 6 vomitted study medication and repeat dose and were given rescue medication and 2 assigned to CD received AL in error, leaving 214 cases, and 206 controls. Parasite DHFR genotyping data was obtained from the three polymorphisms (codons 51, 59 and 108) for 181/214 cases and 170 controls frequency matched on centre. 161/181 (89%) cases were positive for the triple DHFR resistant allele (51I, 59R, and 108N) when enrolled compared to 146/170 (86%) among controls (odds ratio 1.3 (95%CI 0.7–2.5) P = 0.404). Considering parasitological failures by day 14, 51/54 (94%) of treatment failures were positive for the triple DHFR mutation compared to 146/170 (86%) among controls, odds ratio adjusted for centre 3.0 (95%CI 0.85–10, P = 0.088). 111 of the parasitological failures by day 28 were typed on both the day of screening and the day of failure. On day 0, 97/111 (87%) were positive for the triple mutation, on the day of failure, 134/139 (96%) were positive, a significant increase (McNemar X^2^ 6.25 (1df) p = 0.0124).

#### CYP2C19 genotype

All 214 cases and 206 controls were typed. There was no association between CYP2C19*2 genotype and treatment failure. 28% of cases and 29% controls were heterozygous for the CYP2C19*2 allele (odds ratio for treatment failure compared to wild type 1.06 (0.69–1.62) P = 0.803, and 2.9% and 2.3% respectively were homozygous (odds ratio 0.81 (0.24–2.72) P = 0.731). Similar results were obtained when cases were defined as parasitological failures by day 14.

## Discussion

This study, conducted as far as possible under conditions similar to those of routine use of drug treatments by nurses in rural clinics, showed that CD was less effective than AL, there were more early returns to clinic, and higher incidence of severe malaria cases and severe anaemia after treatment, although the frequency of G6PD deficiency was low. In our study only 17/1069 (1.6%) subjects were A- deficient (1 homozygous girl and 16 hemizygous boys). AL, although given without food and with doses other than the first unsupervised, was well tolerated and highly effective among children from 6 months to 10 yrs of age.

Assessment of the safety of antimalarials is complicated when potential side effects of the drugs are also symptoms of the illness, and is complicated further when the risk of adverse reactions is confined to a relatively small subgroup of patients. CD was developed by a public-private partnership as a low-cost treatment for uncomplicated falciparum malaria [1], [17], [18]. The factory price of CD (US$ 0.09 per course for children and $0.29 for adults) made it an affordable alternative to CQ and SP. The combination of two drugs with short half lives minimised the selection for resistance. Concerns were raised about its safety in severely anaemic and G6PD-deficient patients in a review [19]–[21] which concluded that the information on the safety of CD was too limited to warrant its widespread and uncontrolled use. Dapsone can produce dose-related haemolysis that can be severe in patients with G6PD deficiency, but data about the toxic dose in G6PD deficient subjects were limited, (based primarily on data [22] from 5 G6PD deficient male inmates of Illinois State penitentiary given daily doses over 21 days). Two more recent trials of CD combined with artesunate (CDA), in areas where G6PD deficiency is more common, have clearly shown that there is a substantially increased risk of severe anaemia with CD for G6PD A- patients [8], [9] and for this reason CD was withdrawn and the development of CDA was not taken further [2], [35].

In our study anaemia after treatment was more common with CD, but the low frequency of the G6PD A- genotype limited the power of our study to assess this interaction with genotype. In the normal pattern of G6PD deficiency no evidence of haemolysis is apparent until 48–72 hours after substance ingestion [23], [24], we therefore measured haemoglobin concentration on all patients on day 3 after treatment. The nadir of haemoglobin concentration in the trial of Premji *et al.* [8] was at day 7 so it is possible we would have seen more marked effects if we had sampled on day 7. We found evidence of an interaction of treatment group with parasite density, suggesting that failure to rapidly eliminate parasitaemia may have explained the anaemia after CD in G6PD normal subjects.

In sub-Saharan African populations, the most common G6PD deficiency is due to the A- allele with 12% enzymatic activity. G6PD A- is present at high frequencies in many sub-Saharan African countries (e.g. 28 % in Nigeria, [25]). The A- differs from the B (wild type) by two genomic mutations: at nucleotide position 376 A>G with aminoacid change 126 Asn>Asp (G6PD A allele with 85% enzymatic activity), and at nucleotide 202 G>A with aminoacid change 68 Val>Met [26]. However it was recently found that in West Africa the A- allele can also occur with a 968 T>C (323 Leu>Pro) mutation; the 968C was initially found in Senegal, attaining 10% frequency in the Serere ethnic group [27]. It has subsequently been reported that the 968C mutation is present in 8% Gambians, which, combined with a 3% frequency of 202A yields an overall A- frequency of 11% [28]. Although in our study we typed only the 202G>A (the 968C mutation was unreported when our study took place), 5 out of 9 G6PD A- hemizygous males had a drop of 2 g/dL or more in haemoglobin concentration by day 3 after taking CD while only 1 out of 6 who took AL did, consistent with conclusion from other studies [8], [9], [22], [29] that dapsone induces haemolysis in G6PD deficient individuals.

The G6PD ARMS assay used in our study was validated on 100 different G6PD 202 genotypes by an RFLP- based assay [15]. Although Guindo *et al.* have reported G6PD 202 frequencies of more 10% in Malinke people (Malian Mandinka) [33] the frequency of this mutation in Gambians is considerably lower, as also confirmed by Clark *et al.* [28]. The difference in frequency of G6PD deficiency between these populations can be explained by several factors, including admixture and drift, given the historical separation of West African ethnic groups, even when derived from a common ancestral population.

Treatment failure rates after CD in our study were high but not dissimilar to those found in other studies, a comparable CD parasitological failure rate, mainly attributed to reinfections, has been reported from Kilifi District Hospital, Kenya, where a randomised trial of CD using a similar study design in children aged 6 months-5 years with uncomplicated malaria resulted in day 28 parasitological failure in 41% (95% CI 33–49) [3]. In that study, a lower dose (chlorproguanil-dapsone 1.2 and 2.4 mg/kg body weight) was used and no adverse events were reported. The multi-centre efficacy trial, using the same dose formulation of CD as we used in our trial documented day 14 parasitological failure in 4% of patients aged 1–10 years compared to 5.8% in our study [6] (patients in that study were followed-up for only 14 days).

The presence of the DHFR triple resistant mutant at day 0 was not strongly associated with CD treatment failure but the high frequency of this mutation, similar to that seen previously [30], limited the scope to detect an association. The component drugs act synergistically to inhibit the enzymes in the folate pathway, DHPS and DHFR thus making CD pharmacodynamically similar to SP. CD was however predicted to exert less selection pressure than SP because of its shorter plasma half-life (chlorproguanil 12.6 h, dapsone 24.5 h) compared with SP (sulfadoxine 116 h, pyrimethamine 81 h).

We found the frequency of the *2 CYP2C19 allele in Gambians to be similar to that in Europeans. The poor metaboliser phenotype is much more common in Asia (approximately 20%) due to an increased frequency of the *2 allele and the presence of another loss of function allele *3 [11]. The *3 allele was not present in our population and appears to be mainly confined to the Far East. It is reassuring to note that genetic variants affecting the activation of the chlorproguanil component of CD do not influence clinical outcome. This may be because the metabolism of heterozygotes is insufficiently impaired to influence levels of the active compound. However it is interesting that we did not see an effect in homozygotes on clinical outcome. This may simply be because of the small numbers in our study, alternatively the drug may be activated by another cytochrome with similar function. Since we do not have drug levels we cannot examine this hypothesis directly. It is perhaps more likely that the dapsone component is responsible for the pariasite clearance in these people. If this were so then it would tend to argue for use of CD in combination with another antimalarial to postpone development of resistance. It is also possible that gain of function alleles such as the recently described *17 [12] are also present in Africa together with previously undiscovered allelic variants. Thus a more detailed genetic analysis of this population exploring all known functional polymorphisms and detecting novel alleles would be important.

The parasitological failure rate of CD in this study was high. Its slow action, evidenced by symptom clearance by day 3, could be due to poor chlorproguanil metabolism but we failed to see an association with the CYP2C19*2 allele. There has been relatively little work on the pharmacogenetics of antimalarial drugs in Africa–much of our information on potentially relevant alleles comes from Europe and Asia. It would be useful to define the full range of genetic variants in this population which could help to inform policy decisions.

One third of patients treated with AL did not complete their course of medication but despite poor adherence to the recommended regimen, AL had excellent effectiveness up to 28 days after treatment. This is in contrast to a study in Papua New Guinea by Schoepflin *et al.* [31] who attributed a high number of treatment failures to poor adherence to complex dosing regimens in combination with insufficient fat supplementation. In our study the first dose was supervised, in line with best practice, although this was not routine in most health facilities in The Gambia. Treatment success in practice may therefore be lower than in our trial. Adherence may be improved by interventions focusing on provider knowledge and behavior [32], and by the use of dispersible formulation of AL [34].This should be combined with a message about prompt treatment-patients had symptoms for an average of nearly 3 days before reporting to the health centre, and mothers may wait until getting home before giving the first dose if there is no supply of drinking water at the outpatient pharmacy.

## Supporting Information

Checklist S1 **CONSORT Checklist.**
(DOC)

Protocol S1 **Trial Protocol.**
(DOC)
