# Supplementary material for: Randomized Trial of Safety and Effectiveness of Chlorproguanil-Dapsone and Lumefantrine-Artemether for Uncomplicated Malaria in Children in The Gambia
Source: PLoS One. 2011 Jun 7;6(6):e17371. doi: 10.1371/journal.pone.0017371 (PMC3110183; doi:10.1371/journal.pone.0017371)
Supplement: Protocol S1 — Trial Protocol. (DOC) [file pone.0017371.s002.doc]

# Medical Research Council Laboratories, Fajara

**Application to undertake a research project**

**__________________________________________________________________________**

#### A Summary Information

**A1 Title of project**

**Randomised trial of safety and effectiveness of Lapdap® and Co-arthemether® for uncomplicated malaria in operational settings in the Gambia**

**A2 SCC Number ……975………………………(To be allocated)**

# Is this a resubmission?………………………………..YES  NO √

For ALL submissions and resubmissions a version number and version date needs to be entered below. Resubmissions may not be considered without this information

**Version 3. May 12, 2004**

**A3 Investigators (Principal Investigator first)**

Please list all investigators and collaborators and attach CV if the principal investigator is unknown to the Committee

| **Name** | **Institution** | **Position** |
| --- | --- | --- |
| Sam Dunyo | MRC Farafenni Field Station | Clinical Epidemiologist |
| Davis Nwakanma | MRC Farafenni Field Station | High Scientific Officer |
| Musa Jawara | MRC Farafenni Field Station | Entomologist |
| Fanta Njie | MRC Farafenni Field Station | Scientific Officer |
| Paul Snell | MRC Fajara | Senior Data Manager |
| Pauline Kaye | MRC Fajara | Data Manager |
| Maimuna Sowe | MRC Farafenni | Data Manager |
| Robin Bailey | MRC/LSHTM | Epidemiologist |
| Harparkash Kaur | LSHTM | Pharmacologist |
| Colin Sutherland | LSHTM | Molecular Biologist |
| Mamo Jawla | DOSH | Head, NMCP |
| Haddy Gaye | DOSH | Head, Brikama Health Centre CCeCCVentre |
| Baba Balajo | DOSH | Head, NBE DHT |
| Pa Secka | Njaba Kunda Health Centre | Chief Nursing Officer |
| Kalifa Bojang | MRC Fajara | Clinical Scientist |
| Ayo Palmer | MRC CIAM | Director of CIAM |
| Tumani Corrah | MRC Fajara | Director |
| Giorgio Sirugo | MRC Fajara | Head, Human Genetics |
| Paul Milligan | MRC Fajara | Head Malaria Programme PPProProgramme |

**Who will introduce the proposal at SCC? Sam Dunyo**

The PI, if present in The Gambia will normally be invited to present the proposal to the meeting

**A4 Location(s) of research**

Please list all the places where the research will take place including field sites or health facilities

MRC Farafenni Field Station; Farafenni AFPRC Hospital and MCH clinic;

Njaba Kunda Health Centre; Brikama Health Centre; London School of Hygiene and Tropical Medicine (for the testing of drug levels in plasma).

**A5 Proposed start date and duration in months.**

Sensitisation and preparation in August, recruitment September to December 2004, follow-up to January 2005, lab work and data processing to August 2005.

**A6 Summary of project, long term objectives and specific aims (not more than 200 words)**

This section is very helpful to the Committees in determining quickly the main features of the study, and should be as clear and concise as possible. It should cover the key objectives and endpoints and, if the project is hypothesis driven, then the hypothesis should be stated here.

Despite worsening drug resistance, treatment policy for malaria still relies on chloroquine in most West African countries. Lapdap is an affordable and effective drug, but patients with glucose-6-phosphate dehydrogenase (G6PD) A- deficiency are more susceptible to the haemolytic effects of the dapsone component of Lapdap, therefore there is a need to evaluate the extent to which the risk associated with the use of the drug in settings without G6PD screening might outweigh the benefits to malaria treatment. Lapdap acts in a similar way to sulfadoxine-pyrimethamine (S-P) but appears effective against certain S-P-resistant parasites, it is therefore of interest to investigate possible association of Lapdap treatment failure with carriage of S-P-resistant genotypes, and the transmissibility of these genotypes after treatment with Lapdap. Artemisinin combinations are recommended by WHO but the alternatives need to be evaluated in operational settings, because compliance, which affects efficacy and the selection of resistant parasites, may be an important factor in discriminating between alternative treatments. Co-artemether is effective in a supervised 6-dose regimen but its effectiveness when doses are unsupervised has not been evaluated. We will evaluate, in operational settings, the safety and effectiveness of Lapdap and co-artemether for treatment of uncomplicated malaria in patients 6months to 10years.

**A7 Confidentiality**

SCC applications will normally be available on the MRC Gambia intranet with access for all senior staff. If for reasons of commercial, ethical or scientific sensitivity you wish to restrict access/circulation to SCC Committee members only please indicate here.

**Restrict access to SCC members?** YES  NO √ 

**A8 Checklist/Signatures**

**Please complete the following checklist and comment as appropriate.** This section is designed to ensure that all the planning steps have been taken that are needed for a successful project and that the resource requirements are appropriately laid out in Section D. For projects at the MRC Laboratories, Programme Heads will help visiting workers, and others preparing proposals at a distance, to ensure liaison with key individuals who need to be consulted locally.

1. **Has the project been discussed and cleared with the institutions in which research will be carried out including health services to which the study will need access?**

Initial discussions have been held with National Malaria Control Programme, AFPRC Hospital Farafenni and the North Bank East DHT. Discussions have been planned with Njaba Kunda and Brikama health centres.

1. **Have all investigators and collaborators given their agreement to take part in the study as described?**

Yes

1. **Have ethical issues been addressed?** Give details in section C**.**

Yes

1. **Does the project require laboratory work, new laboratory procedures, or the riding of motorcycles? Have the safety issues been addressed?** (The Health and Safety Manager will advise on risk assessment)

Yes

1. **Will the project require data and/or materials to be taken out of The Gambia? If so please give details and sign the following statements:**

Plasma samples will be sent to the London School of Hygiene and Tropical Medicine for the testing of drug levels seven days after treatment. If the molecular techniques required for some of the analysis are not established in The Gambia before the study gets underway then filter papers will be analysed in the London School of Hygiene and Tropical Medicine.

The principal investigator undertakes to leave with the Computer Centre data archivist in The Gambia, a complete copy of the data set at the following two time points:

1. After data entry and verification (“raw data sets”)
2. At the point of submission for publication of final report (“analysis data sets”)

**Signed……………Sam Dunyo………………..**

The principal investigator undertakes to leave with MRC Laboratories The Gambia appropriate aliquots of the biological material being taken out of the country

**Signed……………Sam Dunyo…………**

1. **For projects to be carried out at MRC Laboratories:**

**Has the project been discussed with the following support staff as appropriate and resource requests agreed? (Give details where relevant in section D) :**

|  | DATE DISCUSSED | Comment |
| --- | --- | --- |
| Health and Safety Manager |  |  |
| Head of IT/Senior Data Manager |  |  |
| Director of Clinical Services |  |  |
| Scientific Administrator |  |  |
|  |  |  |
| Transport Manager |  |  |
| Finance Manager |  |  |
| Personnel Manager |  |  |
| Director of Operations |  |  |
| Other services – specify |  |  |

**Signature of principal investigator: Sam Dunyo Date: 12 May 2004**

**________________________________________________________________________**

**B Description of Project**

not more than 5 pages covering the following:

**B1 Background**

The background should show the relationship between the proposed study and the present state of knowledge and should reference previous work by the investigators and others. The results of any pilot experiments should be stated.

Malaria remains one of the most common causes of morbidity and mortality in Gambian children. In rural areas of The Gambia, about 1 in 25 children die from malaria before reaching the age of 5 years. Malaria management has relied upon prompt treatment of clinical attacks with chloroquine (CQ), the most affordable antimalarial drug, delivered through the primary health care system. Treatment at Government health centres is free for children under 5 years. Older children and adults pay a token fee of 1 and 5 Dalasis, respectively. Resistance of *Plasmodium falciparum* to chloroquine has become increasingly common since first reported in the Gambia in 1986 (Menon, 1987). In a study in 2002 in Brikama health centre, (unpublished data), 28% of children treated with CQ failed to show an adequate response by day 14. In The Gambia some clinics give CQ combined with sulfadoxine-pyrimethamine, but there has been no change in the Government’s antimalarial treatment policy due to concern about the impact on availability of increased costs. Since CQ resistance is already common, widespread use of SP in combination with CQ is likely to lead to rapid spread of SP resistance; both drugs when used alone lead to a high prevalence of gametocyte carriage among treated patients, so CQ+SP will not prevent transmission of CQ- and SP-resistant parasites from treated cases. CQ is also not useful as a partner drug in combination with other antimalarials (Sutherland et al. 2003). There is therefore an urgent need to evaluate alternative combination treatments. The rational choice of combination treatment depends on safety, tolerability and efficacy, on the characteristics of the treatment for limiting the spread of resistance, and on cost effectiveness. Effectivenss trials in operational settings are important because adherence may vary and this may be important in discriminating between alternative combinations. Combination treatment with artemisinin and a longer-acting partner drug is the strategy recommended by WHO but these combinations are costly. The combination of chlorproguanil (=lapudrine) and dapsone (Lapdap) has been developed as a fixed-dose tablet primarily for the treatment of semi-immune patients in tropical Africa. It is safe and efficacious in controlled trials with supervised doses (Winstanley, 2001; Lang and Greenwood 2003). It has been registered by the UK Medicines and Healthcare Products Regulatory Agency (MHRA) and also by a number of African countries. At a cost (at the factory) of 9 US cents per course, Lapdap is an affordable alternative to CQ. However, since patients with glucose-6-phopsphate dehydrogenase (G6PD) A- deficiency are more susceptible to the haemolytic effects of the dapsone component of Lapdap, it is important to evaluate the extent to which the risk associated with the use of the drug in settings without G6PD screening might outweigh the benefits to malaria treatment.

Lapdap acts in a similar way to SP, by inhibition of the enzyme dihydrofolate reductase (DHFR). Resistance to pyrimethamine is conferred by simple point mutation of the gene for DHFR (Plowe et al, 1996). It is likely that there is a small but constant rate of mutation of this gene during the parasite’s sexual cycle. Only those mutations conferring benefit surviving to predominate. In the presence of selection pressure of antifolate drug, mutations of DHFR which reduce sensitivity to the drugs tend to be selected during the asexual cycle in the human host, and then pass on to the next host via the mosquito vector. Four point mutations are particularly important, at codons 108, 51, 59 and 164. In Africa the DHFR drug resistant phenotype is initiated by mutation at 108, followed by mutations at 51 and 59; the triple mutant 108+51+59 is associated with poor clinical response. All three mutations occur in The Gambia but their prevalence is not known. Lapdap appears effective against some SP-resistant parasites and is likely to exert less selection pressure than SP because of its shorter half-life. It is therefore of interest to examine possible association of these mutations with Lapdap treatment failure and also to measure the frequency and transmissibility of these genotypes after Lapdap treatment.

Co-artemether is a fixed-dose combination that has been shown to be safe and efficacious in supervised trial conditions. Following findings that the 4-dose regimen produced only 76% cure rates by day 28 in Thailand, compared to 97% for the 6-dose regimen, WHO are recommending the 6-dose regimen. Since compliance to this complex regimen may be poor in operational settings it is important to evaluate coartemether when doses are unsupervised. We therefore propose to compare the effectiveness of Lapdap and Co-artemether for treatment of uncomplicated malaria in an operational setting. We will also investigate acceptability of the treatments and compliance, the safety of treatment in G6PD-deficient and normal malaria patients, the effects of treatment on transmissibility of P.falciparum, the cost effectiveness of the two treatments.

**B2 References**

Lang T and Greenwood B (2003) *Lancet Infect Dis* 3:162-8

Menon A, Snow RW, Otoo L & Greenwood BM (1987). *Lancet*, 11:1029-1030

Müller O, Boele van Hensbroek M, Jaffa S. et al. (1996). *Trop Med Int Health*, **1,** 124-132

Plowe, CV et al. (1995) Am J trop. Med. Hyg 55:467-471.

Ruwende C et al. (1995) *Nature* 376:246-9.

Sirugo G et al. (in press) *American Journal of Human Genetics.*

Sutherland S, Drakeley C, Obisike U et al. (2003) *Am J Trop Med Hyg,*  **69,** 19-25.

Targett G, Drakeley C Jawara M.*et al.* (2001) *J infect Dis*, **183,** 1254-1259.

von Seidlein L et al. (1998) Am J Trop Med Hyg 58:638-644

von Seidlein L et al. (2000) *The Lancet* 355:352-357

von Seidlein, L et al. (in press) *Trans. Roy. Soc. trop. Med. Hyg.*

Watkins WM & Moboso M (1993). *Trans. Roy. Soc. trop. Med. Hyg.* **87,** 75-79

Winstanley P (2001). *Trop Med Int Health,* **6,** 952-954.

### B3 Project description

This should cover project plan, time-scales, descriptions of methods, justification, analyses to be carried out, expected outcomes (see also B4 and B5 where particular details need to be set out and cross refer as necessary).

**Study design and methods**

*Study area and study population*

Patients will be recruited from three health centres, Farafenni Maternal and Child Health (MCH) Clinic, which serves the rural town of Farafenni; Njaba Kunda, a mission clinic in a rural village; and Brikama Health Centre, a Government health centre serving a large urban community. Children aged 6 months to 10 years presenting at health centres with a history of illness, who have a fever or recent history of fever, will be screened and those with uncomplicated malaria, a positive blood smear with a parasite density 2000 to 200,000 parasites/μl, monoinfection with *P. falciparum*, and a packed cell volume of >=20%, will be invited to enrol into the study after written informed consent is given

*Treatment and follow-up*

The study patients will be randomised to receive three daily doses of Lapdap, or a six-dose course of co-artemether. Dosages will be determined according to manufacturers’ instructions. The first dose will be given by the mother under direct observation of the dispensing nurse, and subsequent doses will be given at home unsupervised. The parent/carer will be encouraged to bring the child back to clinic if the child’s condition does not improve

The parent/carer will be visited at home a day after the last dose should have been taken (Day 3) in order to check any left over medication, to ask about compliance and adverse reactions and to collect finger prick blood samples for haemoglobin check using Hemocue (portable haemoglobin analyser machine. Haemoglobin results will be entered in lab forms pre-printed with Day 0 haemoglobin results. Patients with severe anaemia (Hb ≤5 g/dl) or Hb that has fallen by 2g/dl or more from the Day 0 measurement will be promptly referred to the clinic for treatment. Nurses and drivers will be placed on 24-hour emergency duty at the health centres/hospital where the study is sited to handle emergencies. Recruitment will be limited to 5-8 kilometre radius of the study site for quick access to a clinician in the case of an emergency.

The parent/carer will be requested to return the patient to the clinic on day 14 and day 28 after treatment for clinical evaluation and blood sampling for PCV, microscopy and molecular analysis. In one centre, Farafenni, post-treatment transmission of resistant genotypes of *P.falciparum* will be evaluated in transmission experiments, by membrane feeding of post-treatment samples to mosquitoes. In addition to randomization for treatment, subjects in Farafenni will be randomized to be checked for gametocytaemia on either day 7 or day 14 after treatment. Subjects who are gametocyte positive will be asked to give a venous blood sample for mosquito feeds. Samples will be used for feeding to 5-day-old *Anopheles gambiae s.s.* via an artificial membrane, and mosquito midguts dissected 7-8 days later to determine the number of malaria oocysts. The protocol allows assessment of the possible effects of drug or antibodies in the blood meal on transmission. DNA will be extracted from oocysts to determine the presence of genotypes associated with resistance to sulfadoxine and pyrimethamine.

From the finger-prick blood samples collected on filter paper at screening and from samples taken from parasitological treatment failures, 10-20μl aliquots will be tested using nested PCR on extracted DNA to detect point mutations in genes associated with resistance to sulfadoxine-pyrimethamine DHFR (codons 51, 59, 108) and DHPS (codons 437, 540), in order to examine their possible association with Lapdap treatment failure. In addition, if a child has a second episode of parasitaemia, samples from the first and second episode will be assayed for the three sequence families of MSP1 block 2 repeat region and the two sequence families of the MSP2 repeat region, to distinguish recrudescence from reinfection. Since patients with glucose-6-phosphate dehydrogenase (G6PD) A- deficiency are more susceptible to the haemolytic effects of the dapsone component of Lapdap, in this trial we will determine the G6PD genotype using an ARMS PCR technique. Retrospectively, we will screen for the two A- associated mutations, an A to G transition at position 376, encoding the B (normal) to A change (Asn126Asp), and a G to A transition at position 202, encoding the specific A- change (Val68Met), and we will also measure enzymatic activity, in order to evaluate the extent to which the risk associated with the use of the drug in settings without G6PD screening might outweigh the benefits to malaria treatment.

In addition we will measure concentrations of drug components in a sample of day 0 samples and in day 7 samples, using the HPLC method at LSHTM, as a verification of compliance with treatment. An economic component to the studies will be incorporated to assess provider costs as well as social costs associated with treatment and with treatment failure. Qualitative research techniques will be used to investigate mothers’ perceptions of antimalarial treatment and operational issues.

*Endpoints, sample size, randomization, and data management*

The primary endpoint will be clinical and parasitological cure by day 28. Secondary endpoints include the incidence of clinical anaemia at any time during the 28 day follow up, defined as clinical signs of anaemia and Hb that has fallen by 2g/dl or more from the day0 measurement; and change in Hb from day0 to day 28; and percentage transmission on day 7 and day 14 after treatment.

The trial will not be blinded, but the randomization procedure will ensure concealment of the allocation sequence. Databases will be designed in Access.

A Data Safety Monitoring Board will being convened for the trial. The local safety monitor is Dr Ousman Nyan.

# *Description of current infrastructure and resources available for the study*

The MRC’s Field Station in Farafenni has carried out a series of trials since 1998 to determine the safety, efficacy/effectiveness, gametocyte prevalence, density and infectivity to mosquitoes of treatment of uncomplicated malaria with various combinations of antimalarials. A stable colony of A. gambiae mosquitoes has been established in the insectary at the Field Station and these will serve as the source of adult mosquitoes for the feeding experiments. In 2003 we completed a large trial of the effectiveness of three alternative drug combinations for treatment of acute uncomplicated malaria in the same three centres. We propose to adopt a similar design and recruit in the same centres in 2004 for the Lapdap trial, using the same trained staff and equipment. Molecular methods for drug resistance typing are being established in the malaria laboratories in Farafenni and Fajara. G6PD genotyping is established in Dr Sirugo’s laboratory in Fajara.

**B4 Details of study design and investigations**

This section is designed to give the Committees sufficient information to see clearly and quickly the scientific and ethical aspects of the study design. Some parts will not be relevant to all studies. For studies at MRC Laboratories, the please discuss data management arrangements with the Head of Computing. For clinical studies, please discuss the clinical service commitments with the Clinical Director. If you have questionnaires or consent forms prepared, please attach these to the application.

1. **What type of study design is proposed (eg case control, prospective cohort, randomised controlled trial, etc)**

Randomised controlled trial

1. **What is the proposed size of the study (this may relate to patients, cases, controls, survey subjects, laboratory samples etc, as appropriate).**

We require at least 1180 patients to be randomized, this allows for 10% loss to follow up.

1. **Please describe the statistical considerations and sample size calculations involved in determining the size of the study.** (If you do not have access to statistical advice, please consult the MRC Laboratories Statistics Department.)

The sample size is based mainly on the primary endpoint but takes account of a number of other objectives as well. We wish to be able to detect clinically important differences in efficacy, but in the case that both treatments appear similar in efficacy, we will want to be able to rule out the possibility that one of the treatments may be worse, so it is important that the difference in treatment failure rates is estimated with sufficient precision (non-inferiority). We wish to demonstrate that the new combinations are at least as good as each other within a margin of 3%. To have at least 80% power for this, using the method of Farrington and Manning and a significance level of 0.025. The trial will be part of a multi-centre study to be sponsored by WHO. We will pool data from all participating countries for analysis of impact of G-6-PD deficiency on anaemia

1. **For studies involving human subjects. How and where will the study subjects (cases, controls, etc) be selected? Has it been confirmed that they are not already involved in other studies?**

The study subjects will be children presenting to Farafenni MCH clinic, and Njaba Kunda and Brikama Health Centres with a febrile illness and a blood film confirms they have significant parasitaemia. There is ongoing demographic surveillance system in the Farafenni area but this does not preclude enrolment into a clinical trial.

1. **What inclusion/exclusion criteria will be applied?**

The following will be included in the study:

- Children aged 0.5-10 years of age presenting with symptoms of acute malaria infection
- Axillary temperature of ≥ 37.5°C
- Positive blood smear with monoinfection with *P. falciparum*
- Parasite density of 2000-200 000 parasites/μL of blood
- Packed cell volume (PCV) ≥20%
- Free and informed consent of their parents or legal guardians

The following children will be excluded:

- Those with symptoms and signs of severe and complicated malaria
- Severe malnutrition, defined as 3SD below weight for height
- Clinically evident concomitant disease
- History of allergy to the study medications
- Those with a potential problem with follow-up, e.g. if a trip outside the study area is planned before the end of the follow-up period.

1. **What samples, if any, will be taken and what investigations will be conducted?**

- Finger-prick samples on days 0, 7, 14 and 28 and on any day that a study patient returns sick to hospital for thick films for malaria parasite detection and filter paper blood spots for molecular typing and packed cell volume (PCV)/haemoglobin determination.
- 2-3 mL blood by venepuncture on day 7 (MRC Camp in Farafenni only) from consenting gametocyte positive patients for drug level determination and membrane feeding of mosquitoes
- Alcohol-fixed oocysts from infected mosquito guts for genotyping

1. **Will treatment be given? YES √ NO **

**If yes:**

**Nature of treatment(s)**

1. Co-artemether (artemether plus lumefantrine) is an artemisinin-based combination. The lumefantrine component is not fully absorbed unless accompanied by a fatty meal.
2. Lapdap (chlorproguanil; Paludrine) plus Dapsone is a synergistic antifolate drug combination.
3. Paracetamol dispensed as 100mg tablets

**For drugs: dosage and duration of treatment**

Co-artemether is administered twice daily for three days according to the manufacturer’s instructions as follows: children <15 kg 1 tablet; 15-24 kg, 2 tablets; 25-34kg, 3 tablets.

The dosage for Lapdap is chlorproguanil 2.0mg/kg and Dapsone 2.5mg/kg daily for three days.

Paracetamol will be administered as 10 mg/kg three times daily for three days or until fever subsides.

**Person(s) responsible for administering treatment**

The first dose of medication will be administered by an experienced nurse who will serve as the drug dispenser throughout the study, subsequent doses will be given by paraents/care givers at home without the supervision of project staff.

1. **For questionnaires/interviews, who will be conducting these?**

MRC field assistants and nurses (seconded from the health facility where the study is sited) will conduct the initial interview about symptoms. Clinicians will be responsible for clinical interview of all laboratory confirmed cases and those returning sick. MRC field assistants will administer informed consent questionnaires and all follow-up interviews.

**B5 Data management and Statistical analysis**

###### Who is responsible for the statistical design and analysis of the study?

PI/Paul Milligan

1. **Who will be primarily responsible for database design and data management?**

Paul Snell and Pauline Kaye (database development) and Maimuna Sowe (data management).

1. **Will data be double entered and verified? Yes**

e) The MRC Laboratories IT/Data Management section supports Microsoft Access as its database package. If you are planning to use something else, please indicate which package and give a brief rationale:

**B6 Expected outputs and Dissemination of results**

**(a) What are the expected outputs(publications) from this project?**

The study will generate publishable data in the following areas:

Data to document, for the first time, the effect of Lapdap on the transmission potential of drug resistant parasites, and a comparison of these results to our previous work with other antimalarial regimens. Information on the tolerability, practicability and effectiveness of these recommended drug combinations in an African outpatient setting. Such data is urgently needed to inform African policy makers in their decision to switch over to the intervention drugs as first-line treatment in line with WHO expert panel recommendation.

**(b) What other arrangements will there be to disseminate the findings?**

Meetings will be held with the District Health Team of Farafenni (North Bank East) and Brikama (Western Division) and officials of the Department of State for Health to disseminate the results of our findings. The findings will also be presented at an MRC organized seminar and other national and international conferences and workshops. CIAM, NMCP, WHO-RBM and MRC hold regular meetings to discuss common issues, including advice on grant proposals, operational research and policy issues. These trials and their results will thus be made known to the NMCP in a timely fashion and can be incorporated into the ongoing discussions of changes to the recommended malaria treatment. The regional malaria treatment network, RAOTAP, of which The Gambia is a member, will be informed of this study and the results in a timely fashion.

1. **Ethical issues**

This section is particularly important to the Ethics Committee. Please consult the guidance notes for preparation information sheets and consent forms; the checklist for subject information sheets; the template consent form; the guidelines for scientists (EC); the guidelines for the Gambian initiative for DNA Collections, as appropriate.

**(a) Outline how the study will contribute to improving the health of people of The Gambia**

Benefits to trial participants: Both Lapdap and co-artemether are significantly more efficacious than the currently available treatment, chloroquine. All subjects will be reimbursed costs of travel to the health centre and will be provided (free of charge) with appropriate treatment if their condition does not improve or if they become sick during the trial follow-up period.

Subjects will be asked to provide finger prick blood samples at screening and on day 14 and day 28, the screening sample is good clinical practice, the later samples are important to check parasite clearance and haematological recovery.

In addition subjects recruited at Farafenni will be asked for a finger prick sample on day 7 and if this is gametocyte positive, for a venous sample for transmission experiments. Only 2-3 mls will be taken, this is important to determine the drug characteristics for limiting the transmission of resistant parasites. This trial will contribute to the selection of an affordable and effective replacement for chloroquine as first line treatment for uncomplicated malaria.

**(b) Summarise the potential risks and benefits to individuals, communities or country**

Although dapsone (one of Lapdap’s constituents) remains widely used in Africa against leprosy, it produces dose-related haemolysis that can be severe in patients with glucose-6-phosphate dehydrogenase (G6PD A-) deficiency. Anaemia is consequently the most common adverse event associated with Lapdap (Pincock 2003). It is therefore important to evaluate the extent to which the risk associated with the use of the drug in settings without G6PD screening might outweigh the benefits to malaria treatment. In the normal pattern of G6PD deficiency no evidence of haemolysis is apparent until 48-96 hours after substance ingestion. Therefore, the child will be visited at home on day 3 (the day after the last dose of medication should have been taken) by a fieldworker who will ask parent/care giver about the child’s condition, will check for signs of anaemia and haemolysis (pallor and jaundice) and collect finger prick blood sample for haemoglobin analysis. If the child is anaemic or not well he/she will be referred to the clinic for further evaluation and prompt treatment. All participants will have ready access to emergency treatment when adverse events develop. Both Lapdap and the 6-dose regimen of Co-artemether have been shown to be safe and effective under strictly controlled trial conditions but it is not known to what extent poor adherence to the recommended regimens will affect efficacy. Trials in operational settings may help discriminate between the choice of treatment. There are no risks to participating communities or country

**(c) How will informed consent be obtained?**

The study will be explained in the appropriate language to the parents or guardians of eligible children as part of the screening procedure. The objectives and methods, including details of randomisation and treatment allocation, and blood sampling will be explained in the language they understand. The right of individuals not to participate or to withdraw their consent will be respected and the children will then receive standard treatment. Consenting parents/guardians will be requested to sign/thumb-print a written informed consent form as evidence of consent freely given. The recruiting field assistant and the reviewing clinician will sign that consent was given. Parents/guardians may withdraw their consent at any point in the trial and the child’s treatment will not be affected.

**(d) How will you ensure confidentiality of the data gathered?**

The information collected on patients will be treated as confidential. Personal identifiers will not be included in the main database. All data will be entered into the computer and analysed to provide information to answer the research questions only.

**(e) Is a consent form attached?**

Yes

**(f) Is a subject information sheet attached?**

Yes

**(g) Is the questionnaire (if applicable) attached?**

#### D Resources Requested

# D1 Summary and cost

The Committees need to be reassured that the resource implications of the study have been fully considered and that the resources are available or are being sought to complete the study. Please refer to the following guidance in completing the table overpage.

For all cost categories please indicate whether (i)internal funds are requested, (ii) whether you plan to vire uncommitted funds in an existing budget (if so please give budget code and title), or (iii) an external source. If the project has not already been fully costed for a funding application, please discuss the resources needed with the External Grants Coordinator, Finance Manager and the Director of Operations.

As a brief guide:

**Staff**  - please indicate which staff members will be working on the project and the percentage of their time they will commit. If new staff are required please indicate the grade at which the appointment will be made and whether recruitment is internal(existing staff currently on another project) local(The Gambia) subregional(West Africa) or international. For new staff to be employed by MRC, full staff costs (including social security contributions, recruitment etc) may be obtained from the Personnel Manager, or from a spreadsheet operated by the External Grants Coordinator.

**Consumables** – these include all laboratory consumables, medicines and other clinical supplies, questionnaire production, computer consumables, specialist stationery and other supplies particular to the project. Freight costs should be included. The Purchasing Department and Laboratory Manager will advise on costs.

**Access to existing equipment-** Please specify e.g number of computers, and for example access to vehicles

**Capital and minor equipment** – any equipment that needs to be bought, replaced or repaired for the project. Freight and installation costs should be included. For projects at the MRC Laboratories, the Laboratory Manager will advise about the availability of laboratory equipment.

**Laboratory services-** MRC laboratories has facilities for HLA typing, clinical microbiology (including TB) and routine haematology and biochemistry. Please indicate the number of samples to be processed in each area

**Transport** – if MRC transport is requested please indicate approximately how many kilometres of travel will be required. The Transport Manager will advise on the most cost-effective way of meeting the need given the resources available (vehicles, drivers etc). You should allow for local public transport costs for staff or study subjects , and night allowances for staff.

###### Space - indicate the requests for office space, freezer space(including liquid nitrogen storage) and laboratory space implied by the project and how it is suggested that these are met in discussion with the Scientific Administrator

**Conference visits and Presentations-** The Unit is particularly keen to support presentations by higher degree students and scientific officers. Please make the case here

**Other** – this may include, particularly for externally-funded projects, accommodation costs, clinical fees and other overhead charges, communication costs, training, meeting costs, library. Please discuss the availability of residential accommodation with the Housing Manager (short stays) or Director of Operations (longer term) and office space with the Scientific Administrator.

**D2 Resource Request Spreadsheet(available in Excel on request)**

**TIMELINE**

**Please indicate the period of activity of the project**

**August 2004-August 2005**

**NEW STAFF**

| **Grade/Band** | **Where recruited from** | **% time on project** | **Source of funding** |
| --- | --- | --- | --- |
| **Three research clinicians D2** | **W Africa** | **100** | **WHO/TDR** |
| **Scientific officer D1** | **W Africa** | **100** | **MRC** |
| **Molecular Biologist, E1/2** | **W Africa** | **50** | **MRC** |

**EXISTING STAFF**

| **Who(names needed)** | **Grade** | **% time** | **Source of funding** |
| --- | --- | --- | --- |
| **Sam Dunyo** | **E2** | **80** | **WHO/TDR** |
| **Musa Jawara** | **D3** | **60** |  |
| **Fanta Njie** | **D2** | **80** |  |
| **15 Field staff** | **B1-B2** | **100** |  |
| **3 Field supervisors** | **B3** | **100** |  |
| **Senior lab asst** | **B3** | **100** |  |
| **5 Lab assistants** | **B1** | **100** |  |
| **3 drivers** | **B2** | **100** |  |
| **2 Data entry clerks** | **B1-B3** | **100** |  |
| **Pauline Kaye** | **D3** | **10** |  |
| **Maimuna Sowe** | **D3** | **25** |  |
|  |  |  |  |

**OFFICE, LAB, FREEZER SPACE**

| **What** | **Where** | **% use** |
| --- | --- | --- |
| **-70 freezer** | **Farafenni, oocyst samples** | **15** |
| **-20 freezer** | **Farafenni, serum samples** | **15** |
| **Slide reading area** | **Farafenni, 5 people** | **100%** |
| **PCR areas** | **Farafenni** | **70%** |
| **G6PD testing etc** | **Human Genetics Laboratory** |  |

**CAPITAL EQUIPMENT NEEDED >3000 pounds**

| **List:** | **Cost** | **Source of funds** |
| --- | --- | --- |
|  |  |  |

**EXISTING EQUIPMENT TO WHICH ACCESS IS NEEDED**

| **What** | **How often** |
| --- | --- |
| **PCR machine etc in Farafenni** | **70% time** |
| **PCR machine malaria lab, Fajara** | **15% time** |
| **Three portable generators** | **100%** |
| **4 Haematocrit centrifuges** | **100%** |
| **Five compound microscopes** | **100%** |
| **Three dissecting microscopes** | **75%** |
| **Four slide driers** | **100%** |
| **Insectary facilities and membrane feeding equipment** | **80%** |

**NEW MINOR EQUIPMENT REQUIRED**

| **List:** | **Cost** | **Source of funds** |
| --- | --- | --- |
|  |  |  |

**CONSUMABLES**

|  | **What** | **Cost** | **Source of funds** |
| --- | --- | --- | --- |
| **LAB** |  |  |  |
| **Microscope slides** | **10000** |  |  |
| **Slide storage boxes** | **100** |  |  |
| **Sterile lancets** | **10000** |  |  |
| **Gloves** | **100** |  |  |
| **Hliter paper (Whatman No 3)** | **10** |  |  |
| **Giemsa stain (in 1L bottles)** | **10** |  |  |
| **Fields stain (A&B packs)** | **10** |  |  |
| **PCR reagents** |  |  |  |
| **DNA extraction and reagents** |  |  |  |
| **Primers synthesis and restriction enzymes** |  |  |  |
| **Gilson pipettes** |  |  |  |
| **DRUGS** |  |  |  |
| **Study drugs** |  |  |  |
| **Medical care for study patients** |  |  |  |
| **STATIONERY** |  |  |  |
|  |  |  |  |
| **COMMUNICATIONS** |  |  |  |
|  |  |  |  |

**TRANSPORT AND LOCAL TRAVEL**

| **What:** | **Km** | **Cost** | **Source** |
| --- | --- | --- | --- |
| **Land Rover usage** | **400km/week x 24 weeks x 3 vehicles** |  |  |
| **Motorcycle usage** | **150km/week x 24 weeks x 15 bikes** |  |  |
| **Allowances** |  |  |  |
| **Fares** |  |  |  |

**USE OF LABORATORY SERVICES**

| **What:** | **No of specimens** | **Cost** | **Source** |
| --- | --- | --- | --- |
| **HLA Typing** | **N/a** |  |  |
| **Clinical Microbiology** | **N/a** |  |  |
| **Haematology/**  **Biochemistry** | **N/a** |  |  |
| **Serology** | **N/a** |  |  |
| **HIV testing** | **N/a** |  |  |

**CONFERENCE PARTICIPATION**

| **Who/Where** | **When** | **Cost** | **Source** |
| --- | --- | --- | --- |
| **S Dunyo, D Nwakanma, M Jawara** | **MIM Meeting 2005** |  |  |
|  |  |  |  |
|  |  |  |  |

**D3 Sources of funds**

If external funds have been or will be sought, please state the progress of the application(s).

Application for funding submitted to WHO/TDR and EDCTP.

**MRC Lapdap and Co-artemether Transmission Study 2004**

##### Information Sheet

Please explain the following to the mother or guardian of this child:

Your child has malaria, a common and serious disease in Gambian children. There are many drugs that can be used to treat malaria. In the Gambia chloroquine is usually used to treat children with mild malaria and Fansidar is used in some cases. In children who are more sick both drugs are given together and many children have been treated successfully with the combination. Most of the time, a few days after taking chloroquine or Fansidar, the patient feels better and the malaria parasite gradually disappears from their blood. But during this time, if mosquitoes bite the patient then the mosquitoes can become infected with the malaria parasite and give the disease to the next person they bite. Sometimes, these drugs do not work at all since the malaria parasites may be resistant to the treatment. Other options that may be used to treat malaria include Lapdap (a new fixed-dose combination of Lapudrine and Dapsone) and Co-artemether (combination of artemether and lumifantrine). The MRC is carrying out a study to determine if Lapdap or Co-artemether will stop the mosquitoes becoming carriers of malaria. Lapdap and Co-artemether are safe for your child to take.

If you agree that your child can enter this trial, then your child will receive Lapdap or Co-artemether. Treatment allocation will be determined by picking an envelope containing a pre-prepared randomization label generated with the aid of the computer. We will also give your child paracetamol for 3 days. A field worker may visit your house any time during the trial to collect information on the child’s treatment.

We will bring you and your child to the MRC compound in 7 days’ time and a finger prick blood taken. If your child has parasites that may infect mosquitoes we would like to draw a teaspoon (3ml) of blood from your child’s arm to check if he/she is infectious to mosquitoes. To make sure your child is well and has no longer parasites we request you to come to bring your child to the clinic in 14 days and 28 days, for examination and finger prick sampling to check for malaria parasites.

- All the information given to us will be treated as confidential.
- You do not have to let your child enrol in this trial. If your child does join, you can withdraw him/her whenever you wish. Withdrawal will not affect your family’s or your child’s health care. If you do not want your child to be involved in the trial, your child will receive the usual treatment. If you have any questions or queries at any time during the study, the study team will be happy to talk to you about them.

**MRC Lapdap and Co-artemether Transmission Study 2004**

**Consent Form**

**Name of child:……………………………………… Study Number………………**

I have explained the contents of the information sheet in the appropriate language to the study child’s parent/guardian. He/she had the chance to ask questions and I believe that he/she has understood:

That the child has malaria and will be treated with Lapdap or Co-artemether. The child will also be given paracetamol for three days. A field worker may visit their house any time during the trial to collect information on the child’s treatment.

That if the child is unwell at any time during the trial, the parent/guardian should bring him/her back to see the doctor at the clinic for further examination and treatment.

That on day 7, transport will be provided to bring the child and parent/guardian to the MRC Camp in Farafenni. A finger prick will be taken and if the child has the malaria parasites that may infect mosquitoes, we would draw a teaspoon (3 mL) of blood from the child’s arm to check if he/she is infectious to mosquitoes.

That he/she will bring the child to the clinic in 14 and 28 days for examination and blood sampling to make sure that the child is well and no longer has malaria parasites.

**Name of parent/guardian:……………………………………………….……………**

# Signature/Right Thumb Print Date|__|__|/|__|__|__|/|__|__|

I confirm that I have explained to the parent/guardian of the above-named child in the appropriate language, that she/he understood what had been said and that she/he agreed freely that the child should enter this trial

**Name of the interpreter: Signature: Date:**

**Name of the investigator: Signature: Date:**

**MRC Lapdap and Co-artemether Effectiveness Study 2004**

##### Information Sheet

Please explain the following to the mother or guardian of this child:

Your child has malaria, a common and serious disease in Gambian children. In the Gambia chloroquine is used to treat children with mild malaria. In children who are more sick chloroquine and Fansidar are given together and many children have been treated successfully with the combination. In many parts of Africa these drugs are becoming less effective, because the malaria parasites have become resistant to them. The MRC in collaboration with the Department of State for Health are carrying out a study to compare the effectiveness of alternative drug combinations.

If you agree that your child can enter this trial, then your child will be treated with either Lapdap (combination of Lapudrine and Dapsone) or Co-artemether (combination of artemether and lumifantrine). The medications are administered as co-formulated tablets. These drugs are safe and are effective against malaria. We will also give your child paracetamol for 3 days. A field worker may visit their house during the trial to collect information on the child’s treatment.

If the child is unwell at any time during the trial, you should bring him/her back to the health centre, where the child will be treated free of charge, and your transport costs will be reimbursed.

To make sure that the child is well and no longer has malaria parasites, the parent/ guardian is requested to bring the child back to the clinic in 14 days time and again in 28 days’ time for clinical examination and collection of finger prick blood sample for laboratory tests.

- All the information you give to us will be treated as confidential.
- You do not have to let your child enrol in this trial. If your child does join, you can withdraw him/her whenever you wish. Withdrawal will not affect your family’s or your child’s health care If you do not want your child to be involved in the trial, your child will receive the usual treatment. If you have any questions or queries at any time during the study, the study team will be happy to talk to you about them.

**MRC Lapdap and Co-artemether Effectiveness Study 2004**

##### Consent Form

# Site:…………………………………………….. Date |__|__|/|__|__|__|/|__|__|

**Name of child:……………………………………… Study Number………………**

I have explained the contents of the information sheet in the appropriate language to the study child’s parent/guardian. He/she had the chance to ask questions and I believe that he/she has understood:

That the child has malaria and will be treated with Lapdap or Co-artemether. The child will also be given paracetamol for three days. A field worker may visit their house any time during the trial to collect information on the child’s treatment.

That if the child is unwell at any time during the trial, the parent/guardian will bring him/her back to see the doctor at the clinic for further examination and treatment.

That in order to make sure that the child is well and no longer has malaria parasites, the parent/ guardian will bring the child back to the clinic in 14 and 28 days’ time for clinical examination and collection of finger prick blood sample for laboratory tests.

**Name of parent/guardian:……………………………………………….……………**

# Signature/Right Thumb print

I confirm that I have explained to the parent/guardian of the above-named child in the appropriate language, that she/he understood what had been said and that she/he agreed freely that the child should enter this trial

**Name of the interpreter: Signature: Date:**

**Name of the investigator: Signature: Date:**
